# Metric System in the United States Army

**Published:** 1878-06

**Authors:** 


METRIC SYSTEM IN THE UNITED STATES ARMY.—The last circular, issued by Surgeon-General Woodworth, of the Marine Hospital service, stated that medical officers of the Marine Hospital service will hereafter, for all official, medical, and pharmacal purposes, make use of the metric system of weights and measures. In expressing quantities by weight the terms "gramme" and "centigramme" only will be used, and in expressing quantities by measure the term "cubic centimetre." The metric system has already, under the act of July 28, 1866, been adopted by this service for the purveying of medical supplies, and the weights and graduated measures, as well as the glassware, hereafter furnished the medical officers will be in accordance therewith.

Simple rules for the ready conversion of terms of the United States apothecaries' weights and measures into their respective equivalents in metric terms are appended, which, for all medical and pharmacal purposes, are believed to afford sufficiently accurate results. Suggestions are also given as to the mode in which metric medical prescriptions might be constructed, and in relation to the preparation of requisitions for medical supplies in metric terms.—*The Clinic.*

